# Artificial Intelligence Applications in Oral and Maxillofacial Surgery—A Narrative Review

**DOI:** 10.3390/dj14100668

**Published:** 2026-10-13

**Authors:** Yifat Manor, Lir Abergil, Shoshana Reiter, Gil Ben-Izhack

**Affiliations:** 1 Department of Oral and Maxillofacial Surgery, School of Dental Medicine, Gray Faculty of Medical and Health Sciences, Tel Aviv University, Tel Aviv 6997801, Israel; 2 Dental Department, Shamir Medical Center, Beer Yaacov 70300, Israel; gilbenizhack@gmail.com; 3 School of Dental Medicine, Gray Faculty of Medical and Health Sciences, Tel Aviv University, Tel Aviv 6997801, Israel; lirabergil@mail.tau.ac.il; 4 Department of Oral Pathology, Oral Medicine and Maxillofacial Imaging, School of Dental Medicine, Gray Faculty of Medical and Health Sciences, Tel Aviv University, Tel Aviv 6997801, Israel; shoshana.reiter@gmail.com

**Keywords:** artificial intelligence, oral and maxillofacial surgery

## Abstract

**Background**: The use of artificial intelligence (AI) is rapidly growing in the field of dental medicine and in oral and maxillofacial surgery (OMS). The aim of the present review is to assess current applications of AI in OMS, to evaluate their advantages and limitations, and to identify areas requiring further research. **Materials and Methods**: A comprehensive search was conducted using PubMed, Scopus, and Google Scholar, with targeted keywords including “artificial intelligence,” “deep learning,” “machine learning,” “oral surgery,” “maxillofacial surgery,” and “diagnosis “Artificial Intelligence” OR “Machine Learning” OR “Deep Learning” AND “Oral and Maxillofacial Surgery” OR “Oral Surgery” OR “Maxillofacial Surgery” AND “Diagnosis”. Of 308 identified studies, 37 met the inclusion criteria focusing on AI-assisted detection interpretation and surgical planning in OMS. **Results**: AI is effective in identifying anatomical landmarks, evaluating lesions and cysts, diagnosing bone disease like medication-related osteonecrosis of jaws (MRONJ), planning dental implants, and aiding in the classification of fractures and temporomandibular joint (TMJ) disorders. AI can match or outperform human experts in various diagnostic tasks, particularly in panoramic and cone-beam computed tomography (CBCT) image analysis. AI shows promise in early detection of impacted and supernumerary teeth, maxillofacial pathologies, neoplastic conditions, and soft tissue calcifications. Limitations: However, substantial methodological heterogeneity, inconsistent reporting of AI performance metrics, and limited external validation across the included studies constrain direct comparison of results and assessment of the generalizability of reported AI performance. **Conclusions:** AI may assist clinicians in diagnostic and treatment-related decision-making, but current evidence does not support its use as a replacement for comprehensive clinical assessment and professional judgment.

## 1. Introduction

Artificial intelligence (AI), a term first coined by John McCarthy [1], refers to machines that can imitate, induce and improve human knowledge and behavior. Computer systems and machines have been developed that can perform tasks that typically require human intelligence, including problem-solving, learning from experience, recognizing patterns, understanding natural language, and making decisions. AI systems may enhance and refine data by processing and analyzing vast amounts at speeds and scales far beyond human capabilities. Machine learning was first mentioned in 1959 by Arthur Samuel [2] who defined it as a process that enables computers to learn without being explicitly programmed. Today, machine learning enables computers to classify data and predict outcomes based on vast datasets. This capability was significantly enhanced with the advent of deep learning, which revolutionized data processing by allowing computers to handle complex algorithms efficiently. The integration of graphics processing units (GPUs) further accelerated deep learning, enabling faster and more sophisticated computations [3].

In classical machine learning, the features are first engineered by human experts and then learned (e.g., regression modeling). In deep learning, relevant features are learned and mapped in one step, without human feature engineering; this allows leverage of even complex data structures like imagery or language [4]. A deep convolutional neural network (DCNN) is a type of artificial neural network specifically designed to recognize patterns in data, particularly in tasks related to images and visual information.

There has also been a significant uptake of these technologies in medicine, so far mainly in the field of computer vision. A number of drivers for this uptake have been identified [5]:

(1) Diagnostic imaging is central in many healthcare fields, with AI being especially suitable to overcome variability in individual examination and to increase the effectiveness of care while lowering costs by eliminating routine tasks.
(2) Digital health data are collected and organized in a cleaned, curated structural manner.
(3) AI enables integration of different and heterogeneous data domains, for example, medical/dental history, sociodemographic and clinical data, imagery data, biomolecular data, social network data, etc., thereby making the best use of these multi-level data and enabling their interactions to be grasped.
(4) AI may streamline routine work and increase the face-to-face time which doctors/dentists have with their patients (“humanizing care”). This may not only come via diagnostic assistance systems, but also through voice, speech, and text recognition and translation, enabling doctors/dentists to reduce the time required for record keeping [6].
(5) AI enables self-monitoring and self-management by patients who can provide data through use of wearables.
(6) Using these continuously collected data may also overcome the disadvantages of “on-off-medicine” [7], where patients are seen only for a few minutes, while most health conditions are usually acquired over years. Continuous non-invasive monitoring of health and behavior will enable a much deeper, more individual understanding of the drivers and processes underlying health and disease.
(7) Diagnostic and treatment costs may be decreased, thereby relieving healthcare systems burdened by an ageing society with increasingly high numbers of complex, chronically ill cases. AI may also help to address shortages in workforce, as are presently observed and expected to continue in many parts of the globe, thereby supporting the World Health Organization (WHO)’s Sustainable Development Goals [8].

In dentistry, imaging is an integral component of diagnosis and treatment planning. AI can assist clinicians in image interpretation; however developing reliable AI models requires large diverse data sets that encompass a broad range of anatomical and pathological features. These factors are essential for accuracy capturing the underlying structure and variability of the data. Larger, more diverse datasets reduce the model’s susceptibility to random noise and sampling bias, improving its robustness, reliability and generalizability.

In deep learning, models with large numbers of parameters (such as neural networks) require substantial amounts of data to learn effectively. This is because they have a high capacity for learning complex relationships.

High-resolution medical images often exceed memory limits, requiring preprocessing to make them suitable for deep learning applications. Two common solutions are available. The first is image resizing, which reduces memory load but causes loss of resolution and of fine details. The second is segmentation, where the region of interest (ROI) for 2D or 3D images is cropped out to preserve clinically relevant details without overloading memory [9].

To address data scarcity and improve generalization, data augmentation is widely used. This process applies transformations (e.g., rotation, scaling, contrast changes) to existing training images, effectively enlarging the dataset and simulating variations in acquisition parameters and patient anatomy. Data augmentation serves as a form of regularization that reduces model overfitting and enhances robustness [10].

The dataset is typically divided into training, validation, and testing subsets. The training set is used to optimize network parameters across multiple epochs. The validation set (optional) helps fine-tune algorithms by optimizing parameters, while the test set evaluates the final model on previously unseen data to confirm performance [10,11].

Performance evaluation relies on quantitative metrics, including accuracy, area under the curve (AUC) and F1-score, as well as overlap indices [4]. In addition, sensitivity (true-positive rate) measures the model’s ability to correctly identify individuals with a condition, while specificity (true-negative rate) assesses its ability to exclude individuals without the condition. Together, these metrics provide a comprehensive assessment of diagnostic reliability.

The aim of this narrative review was to evaluate current applications of artificial intelligence (AI), machine learning (ML), and deep learning (DL) in oral and maxillofacial surgery (OMFS), with particular emphasis on diagnostic imaging, anatomical structure identification, surgical planning, prediction of clinical and surgical outcomes, and disease assessment. The review also aimed to summarize the potential advantages and limitations of these technologies and to identify current gaps and priorities for future research and clinical implementation.

## 2. Materials and Methods

In order to identify relevant studies related to the role of artificial intelligence in the field of oral and maxillofacial surgery, a comprehensive literature search was conducted. Databases of PubMed, Scopus, and Google Scholar were searched systematically from the inception of each database, with an initial search covering the period up to 31 December 2024. An updated search was then conducted on 31 December 2025, covering publications through 1 December 2025.

Search terms included combinations of “artificial intelligence,” “deep learning,” “machine learning,” “oral surgery,” “maxillofacial surgery,” and “diagnosis”. Studies were eligible if they evaluated an artificial intelligence, machine learning, or deep learning application relevant to oral and maxillofacial surgery, including diagnostic assessment, anatomical structure identification, surgical planning, disease classification, and prediction of clinical or surgical outcomes. AI applications represent a substantial component of the available OMS literature, and they were intentionally included because imaging plays a central role in diagnosis and surgical planning. Only English-language studies related to artificial intelligence applications in oral and maxillofacial surgery were included. Furthermore, an examination of the reference lists of each article was carried out in order to identify other relevant information. Studies were excluded if they were unrelated to oral and maxillofacial clinical practice, did not evaluate an AI/ML/DL application, were case reports, were narrative or editorial publications without original data, or did not provide sufficient information regarding the AI application or its clinical/diagnostic performance. Given the nature of the study, IRB approval was not applicable.

### Risk-of-Bias Assessment

A structured qualitative assessment of methodological quality and potential risk of bias was performed for the included studies. The full ROBINS-I tool was not formally applied. Instead, predefined evaluation criteria were developed based on methodological principles informed by ROBINS-I and TRIPOD/TRIPOD-AI, adapted to the characteristics of AI-based studies in oral and maxillofacial surgery.

The assessment focused on four principal domains: (1) selection bias, (2) ground truth definition, (3) risk of overfitting, and (4) validation strategy. Selection bias considered the study population and dataset selection and their potential representativeness. Ground truth assessment considered the definition and reliability of the reference standard used for model development and evaluation. Overfitting risk considered factors such as dataset size, model complexity, data partitioning, and the methods used to reduce or assess overfitting. Validation strategy considered the manner in which model performance was evaluated, including the use of independent test datasets and external validation.

An overall methodological assessment was additionally assigned based on the combined concerns identified across these domains. Each domain was qualitatively classified as indicating low, moderate, or high concern, and the findings were summarized using a traffic-light system in Table S3. The traffic-light ratings therefore represent an adapted qualitative assessment and not a formal ROBINS-I assessment.

## 3. Results

This study was conducted as a structured narrative review guided by the principles of the PRISMA 2020 Statement. The review aimed to evaluate current applications of artificial intelligence (AI), machine learning (ML), and deep learning (DL) in maxillofacial surgery applications, with emphasis on diagnostic imaging, surgical planning, prediction models, and radiographic interpretation.

A total of 308 articles were identified from a search of electronic databases and a manual search.

**Figure 1:**
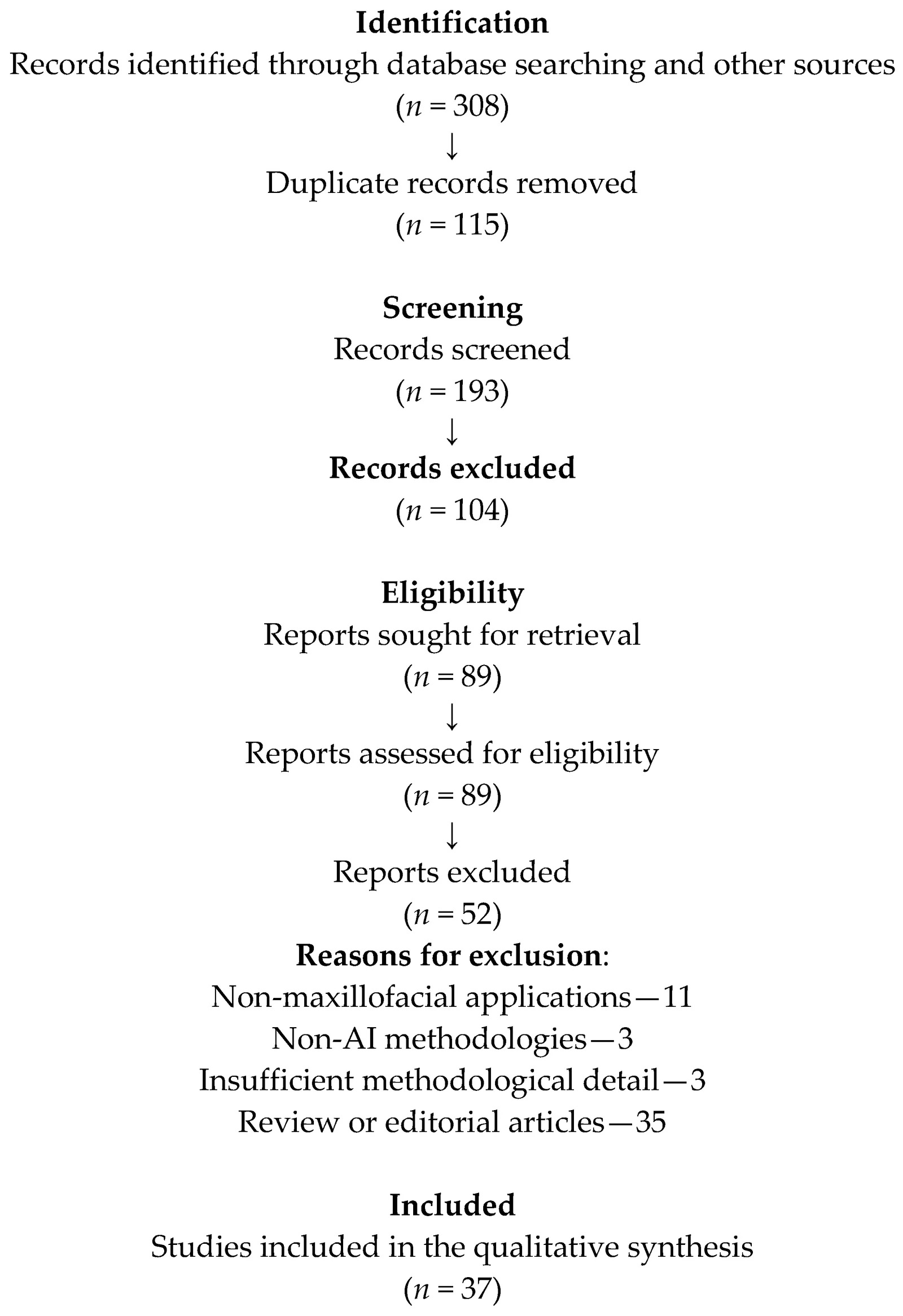
PRISMA 2020 flow diagram of the study selection process.

Out of 308 identified studies, 37 met inclusion criteria focusing on AI-assisted imaging interpretation and surgical planning in OMS. A total of 115 articles were removed due to duplication. A total of 193 were identified for title and abstract screening. Of these, 104 did not meet the inclusion criteria after abstract screening. As a result, 89 were identified for title and abstract screening and fully reviewed. Of these, 37 met the inclusion criteria (Figure 1).

The chosen articles were summarized in a data table (Table S1) according to the following different oral and maxillofacial surgery (OMS) topics:

(1) Identification of anatomy.
(2) Dental implants.
(3) Jaw fractures.
(4) Lesions in the jaws.
(5) Maxillary sinus diseases.
(6) Impacted teeth + mesiodens.
(7) MRONJ medication-related osteonecrosis of jaws.
(8) Osteoarthritis (OA) + TMJ (temporomandibular joint).
(9) Others, including soft tissue.
(10) Ortho surgery and OSA (obstructive sleep apnea).

Table S2 provides an overview of the included studies and their clinical applications, study design, imaging modality, AI methodology, dataset characteristics, reported performance, and validation strategy. Most studies demonstrated moderate risk of bias due to retrospective datasets and reliance on internal validation. Prediction-oriented studies, particularly those estimating operative duration or postoperative complications, showed higher risk of bias because of increased susceptibility to overfitting and dependence on heterogeneous clinical variables. In contrast, studies involving anatomical site identification and surgical planning exhibited a comparatively lower methodological concern due to clearer ground truth definitions. None of the included prediction studies fulfilled all criteria for low risk of bias, primarily due to lack of external validation and potential overfitting associated with retrospective datasets.

### 3.1. Identification of Anatomy

A retrospective study [12] aimed to determine the relationship between lower third molars and the inferior alveolar canal and their three-dimensional (3D) location bucco-lingually according to panoramic X-ray, and compared the ability of AI and oral and maxillofacial surgeons; it found that the accuracy of AI was higher than that of the surgeons. Another study [13] aimed to explore the feasibility of detecting the mental foramen (MF) in DPRs with pretrained object detection models, and to investigate the possibility of developing a tool for automatic measurement of the MCW (mandibular cortical width). The MF is an important landmark for dental practitioners. Detecting its location on a DPR is the most important step in measuring the MCW, which can be a useful index for osteoporosis screening. In this study, EfficientDet-D0 showed sufficient precision and produced correct predictions of MF locations. However, the algorithm needs improvement in cases of high complexity. The importance of identification of anatomical landmarks is emphasized for young dentists without a lot of experience in the dental field and for those who are less familiar with the anatomy.

AI demonstrated higher performance in anatomical landmark detection than the participating human observers in the evaluated study setting; however, this finding was based on a specific research dataset and should not be assumed to reflect performance across broader clinical settings.

### 3.2. Dental Implants

Dental implant planning: A study investigated the differences between AI and oral and maxillofacial surgeons to evaluate the success of the AI system in implant planning using three-dimensional cone-beam computed tomography (CBCT) images.

In the bone height measurements, there were no statistically significant differences between AI and manual measurements in the premolar region of the mandible or in the premolar and molar regions of the maxilla (*p* > 0.05). Among the bone thickness measurements, there were statistically significant differences between AI and manual measurements in all regions of maxilla and mandible (*p* < 0.001). Also, the percentage of right detection was 72.2% for canals, 66.4% for sinuses/fossae, and 95.3% for missing-tooth regions [14].

Evaluation of a dental implant: A study sought to investigate whether deep learning-based prediction of osseointegration of dental implants using plain radiography is possible. The study found that osseointegration of dental implants can be predicted to some extent through deep learning using plain radiography. This method is expected to complement the other evaluation methods of dental implant osseointegration that are currently widely used [15].

Regarding dental implant fractures [16], it was found that AI showed acceptable accuracy in the detection and classification of fractured dental implants, with the best accuracy performance being achieved by the automated DCNN architecture using only periapical images.

### 3.3. Jaw Fractures

Regarding detection of jaw fractures, it was found that AI can provide a better level of performance than general dentists, especially in the mandible, and can therefore be a useful tool, especially in hospitals [17,18,19].

### 3.4. Lesions in the Jaws

Several studies [20,21,22,23] screened the ability to evaluate detection and diagnosis of three types of odontogenic cystic lesions. It was found that AI can provide a predictable differential diagnosis accuracy between ameloblastoma and odontogenic keratocyst. The role of AI was found to be important to oral and maxillofacial surgeons because it provided them with this utility before surgery, helping them to differentiate between lesions in a short period of time [9].

Oral cancer—a study investigated the ability of AI to predict tumor grade (TG) and nodal status (NS) in oral and pharyngeal cancer. It was found that AI was able to predict TG and NS in patients with oral and pharyngeal squamous cell carcinoma [24].

### 3.5. Maxillary Sinus Diseases

AI was also found to be useful in maxillary sinus diseases, especially for classification of pseudo cysts in the maxillary sinus [25].

AI can diagnose maxillary sinusitis according to panoramic X-rays [26,27].

Regarding the use of AI in the prediction of the risk for oroantral communication during tooth extraction, a study used five algorithms of methods of machine learning on panoramic X-ray. It was found that the AI was effective in identifying patients without OAC; however, its ability to detect all true-positive cases was limited [28,29].

### 3.6. Impacted Teeth

An evaluation of impacted third molar teeth in cone-beam computed tomography (CBCT) images found that the system had high accuracy in detection but not in measurement of relation to anatomical landmarks. This should be improved. It is also recommended that a tool be developed which will predict the potential for tooth eruption and identify any real relationship with the mandibular canal/maxillary sinus [30].

Mesiodens location: A study to identify and locate mesiodens simultaneously in panoramic images developed a model that detects mesiodens in primary, mixed, and permanent dentitions. The developed model has the potential to help dental clinicians diagnose mesiodens on panoramic radiographs. In another work, researchers found favorable performance of AI in supernumerary diagnosis, and the deep learning network models used in this study delivered high accuracy in classifying the presence of mesiodens in mixed-dentition panoramic radiographs. The results of this study are expected to help clinicians make decisions by automatically classifying these teeth on panoramic radiographs taken during the eruption period of the maxillary anterior teeth, thereby reducing complications that may arise from failure of early detection of mesiodens [31,32,33,34,35].

Regarding impacted teeth, it is very important to improve the diagnosis of supernumerary and impacted teeth. AI could facilitate such diagnosis and treatment, especially in children for whom orthodontic treatment is planned.

### 3.7. MRONJ—Medication Related Osteonecrosis of the Jaw

Artificial intelligence (AI) has shown promising potential to enhance diagnosis and management of medication-related osteonecrosis of the jaw (MRONJ). It may serve in predictive models for predicting MRONJ in patients with osteoporosis undergoing tooth extraction or implantation. It was found that machine-learned models can assist in predicting MRONJ occurrence in patients based on questionnaire data acquired at the first visit by those patients [36]. Use of an artificial intelligence platform may assist patients in gaining a fundamental understanding of MRONJ [37]. AI helps to identify the early stages of MRONJ and to analyze the size of the preoperatively calculated defect volume of MRONJ patients based on CT and PET CT. Incorporating it into 3D imaging can assist in preoperative planning by enabling accurate assessment of the extent of necrotic lesions. This aids in determining precise resection borders, thereby optimizing surgical outcomes [38].

### 3.8. AI and TMJ Arthritis

The study by Shoukri et al. [39] investigated the relationships among inflammatory biomarkers, clinical symptoms, and condylar surface morphology in patients with temporomandibular joint osteoarthritis (TMJ OA). Significant correlations were found between specific biomarkers and variations in condylar morphology. Using data from 259 condyles to stage TMJ OA, the predictive accuracies of experts and AI were found to be 73.5% and 91.2%, respectively. However, it should be noted that this result is derived from a single study, that the evidence is limited, and that it may not represent general performance.

Other studies analyzing TMJ morphology in panoramic X-rays and in the presence of TMJ arthritis found favorable performance of AI when contributing to the effectiveness of screening tools for dentists [40,41].

### 3.9. Soft Tissue Calcifications and Others

AI improved the accuracy and reduced the time invested by oromaxillofacial radiology specialists [42] in the detection sensitivity of sialolith and lymph node calcification and of carotid artery calcification. AI showed potential for detection of plaques in the carotid in panoramic X-rays [43]. In another study, AI helped in diagnosis of Stafne defects in panoramic X-rays with high performance [44]. According to this study, AI improves diagnosis in the field of oral and maxillofacial surgery by reducing time and increasing accuracy. However, because this was a retrospective investigation, conducted at a single institution, it has limited independent external validation.

### 3.10. AI and Ortho Surgery and OSA

An AI algorithm demonstrated high accuracy in segmenting anatomical structures and assessing airway dimensions, suggesting its potential as a reliable tool for clinicians in diagnosing and planning treatment for OSA patients [45].

Regarding ortho surgical treatment, the effect of mandibular advancement on soft tissue changes was investigated for the AI contribution and it was found that a deep learning algorithm offers a more precise and clinically acceptable method for soft tissue prediction in orthognathic surgery [46].

A study regarding planning of mini-implants location for orthodontic treatment found that an AI system could be valuable in determining optimal sites and designing surgical guides for palatal orthodontic mini-implants [47]. In this field also, the contribution of AI is emphasized.

In summary, in several studies AI demonstrated favorable performance comparable than to that of human observers. However, these results were obtained in controlled research settings and may not directly reflect performance in routine clinical practice.

### 3.11. Observed Risk of Bias

Several studies demonstrated a moderate to high risk of bias. The risk-of-bias assessment revealed methodological concerns across several studies, particularly regarding participant selection, ground truth definition, overfitting, and limited independent validation (Table S3).

## 4. Discussion

The abilities of AI and its performance in the identification of critical anatomical structures are important to all dentists and young experts. Although several studies included in this review reported high AI performance, most were retrospective investigations, frequently conducted at a single institution, with limited independent external validation. Accordingly, performance observed under controlled research conditions should not be assumed to translate directly to heterogeneous real-world clinical environments. Differences in patient populations, disease prevalence, imaging acquisition parameters, scanner characteristics, image quality, and clinical workflows may substantially affect model performance and generalizability. Furthermore, reported performance exceeding that of human observers in individual studies should be interpreted as study-specific performance within the evaluated dataset rather than as evidence of general clinical superiority. At present, AI should be regarded primarily as a decision-support tool that may assist clinicians in diagnostic assessment, treatment planning, and risk stratification, while final clinical decisions remain dependent on professional judgment and the broader clinical context. Prospective studies and independent external validation across diverse institutions and patient populations are therefore required before widespread clinical implementation can be supported.

In the area of MRONJ, AI could contribute to case analysis and decision-making. Early detection remains crucial, particularly in ambiguous clinical scenarios such as periodontal-like presentations or cracked teeth that may actually represent early MRONJ. A study [48] highlighted the use of bone scintigraphy and ^18^F-FDG PET/CT as effective imaging modalities capable of detecting metabolic and bone changes before the onset of clinical symptoms. Integrating AI with these nuclear imaging tools could enhance diagnostic precision, allowing earlier intervention and thus minimizing the severity of MRONJ. Moreover, AI’s ability to process complex data sets may assist in treatment planning, in supporting collaborative clinical decisions and in potentially improving outcomes.

The scope of AI applications in third molar surgery is also expanding beyond image-based detection and classification. Kwon et al. [49] introduced a deep learning model, combining panoramic imaging and clinical parameters, to predict the time required for mandibular third molar extraction. Trachoo et al. [50] demonstrated a deep learning system for predicting the difficulty of impacted mandibular third molar removal using panoramic radiographs, illustrating the potential of AI to assist with preoperative surgical assessment. More recently, Jain and Jadhav [51] developed and validated machine learning models incorporating demographic, radiographic, and intraoperative variables to predict multiple postoperative complications, with explainable AI providing insight into the contribution of individual predictors. Together, these studies illustrate a progression from automated image interpretation toward more comprehensive, patient-specific decision support. AI may help as a decision-support tool that may assist clinicians in diagnostic assessment and treatment planning. The potential applications of AI extend to impacted and supernumerary teeth, particularly in pediatric patients undergoing orthodontic treatment. Early and accurate diagnosis facilitated by AI could streamline treatment planning. It could assist in decision-making for planned surgeries to remove impacted and supernumerary teeth.

AI has also been applied in the detection of rare but clinically relevant entities such as Stafne bone defects, reducing unnecessary imaging and patient anxiety. Additionally, AI-enhanced analysis of panoramic radiographs has shown promise in identifying soft tissue calcifications. Although the detection sensitivity varies by lesion type—with carotid artery calcifications being more easily detected than sialoliths or lymph node calcifications—AI systems can aid in the incidental discovery of atherosclerotic plaques, potentially contributing to broader systemic health assessments.

Collectively, these studies highlight the potential role of AI in various aspects of oral and maxillofacial practice. AI-based approaches may support diagnostic assessment, treatment planning, and clinical decision-making by improving efficiency and assisting in interpretation of complex clinical and imaging data. However, the reported benefits are largely based on study-specific findings, often derived from retrospective datasets, and their generalizability to routine clinical practice remains uncertain. Further prospective studies, external validation, and evaluation in diverse clinical settings are needed to determine the clinical utility of these technologies and their appropriate integration into everyday workflows.

The presence of methodological heterogeneity and of variable reporting standards across the included studies highlights the ongoing need for standardized frameworks in AI research. A particularly important limitation is the frequent reporting of classification accuracy as the sole performance metric. Accuracy alone may provide a misleading or overly favorable impression of model performance, particularly in class-imbalanced datasets in which a model may achieve high overall accuracy by correctly classifying the more prevalent class while performing poorly in identifying the less frequent but more clinically important class. Therefore, accuracy should not be interpreted in isolation. Future studies should routinely report complementary performance measures, including sensitivity, specificity, area under the receiver operating characteristic curve (AUC), and 95% confidence intervals, together with the underlying class distribution and, where appropriate, additional metrics suitable for imbalanced datasets.

### 4.1. Explainability, Clinical Integration, Privacy, and Regulatory Considerations

Despite the promising diagnostic and predictive performance reported in the included studies, several important barriers must be addressed before AI systems can be safely and effectively integrated into routine oral and maxillofacial surgery (OMFS) practice. One major concern is the limited interpretability of many deep learning models. These systems may generate highly accurate predictions without providing a clinically understandable explanation of how the prediction was reached. This “black-box” characteristic can make it difficult for clinicians to identify whether a model has based its prediction on clinically meaningful anatomical or pathological features or on unintended image characteristics and dataset-specific artifacts. Explainable-AI (XAI) techniques, including saliency maps, heat maps, feature attribution methods, and other visualization approaches, may improve transparency. However, visual explanation methods such as heat-maps and saliency maps should not be considered equivalent to clinical ground truth, as the highlighted regions may not necessarily correspond to clinically relevant anatomical or pathological features. Their clinical interpretability and reliability therefore require independent validation before they can be used to support clinical decision-making. Explainability methods themselves require validation, and these should be evaluated for consistency, reliability, and clinical usefulness. The World Health Organization emphasizes transparency, explainability, human oversight, and accountability as important principles for the responsible use of AI in health care.

The issue of explainability is particularly relevant in OMFS, where AI-generated recommendations may influence decisions involving anatomical structures at risk, surgical approach, implant placement, or postoperative complication management. A prediction that cannot be adequately interpreted or challenged may be difficult to incorporate into clinical decision-making, particularly when the consequences of an incorrect prediction could include nerve injury, hemorrhage, infection, or other surgical complications. Accordingly, AI systems should be regarded primarily as decision-support tools, with appropriately trained clinicians retaining responsibility for integrating AI outputs with the patient’s medical history, clinical examination, imaging findings, and other relevant information. AI performance demonstrated in a controlled research dataset should therefore not be interpreted as evidence that the system can independently replace expert clinical judgment.

Another important challenge concerns integration into existing clinical workflows. For imaging-based applications, AI systems should ideally be incorporated into established picture archiving and communication systems (PACS) and radiology/imaging workflows, rather than requiring clinicians to manually export images, upload them to separate platforms, and retrieve results through an independent interface. Such additional steps may increase workflow burden, introduce opportunities for data-transfer errors, and reduce adoption. Effective integration requires interoperability with existing imaging infrastructure, appropriate handling of DICOM data and metadata, acceptable processing times, clear presentation of AI findings, and mechanisms for clinicians to review, accept, reject, or override AI-generated outputs. Importantly, AI results should be presented in a manner that does not obscure the original images or create automation bias, whereby clinicians may place excessive reliance on an algorithmic recommendation. Workflow integration should therefore be evaluated not only according to technical accuracy but also according to usability, the effect on reporting or surgical-planning time, the frequency of clinician overrides, and any potential changes in diagnostic or clinical decision-making.

Patient-data privacy and cybersecurity represent additional considerations. AI development frequently requires large collections of radiographic and clinical data, and these datasets may contain identifiable or potentially re-identifiable patient information. Responsible implementation therefore requires appropriate data governance throughout the entire data lifecycle, including data collection, de-identification, storage, transfer, model development, deployment, and subsequent monitoring. Particular attention is required when patient images or clinical information are transferred to cloud-based or third-party AI platforms. Compliance with applicable privacy and data-protection legislation, institutional policies, informed-consent requirements, access controls, encryption, and data-retention policies should be established before clinical deployment. WHO guidance emphasizes protection of autonomy, privacy and confidentiality, together with appropriate governance and accountability throughout the AI lifecycle.

Medico-legal responsibility is another unresolved issue. When an AI system contributes to an incorrect diagnosis, missed finding, inappropriate surgical plan, or inaccurate risk prediction, responsibility may involve multiple stakeholders, including the treating clinician, the healthcare institution, the software developer, and the device manufacturer. The allocation of responsibility may become particularly complex when clinicians are expected to review AI outputs but the system is difficult to interpret; when the clinician overrides an apparently incorrect recommendation; or when a model’s performance deteriorates because of changes in patient populations, imaging equipment, acquisition protocols, or disease prevalence. Clear documentation of the intended use and the limitations of an AI system, as well as appropriate clinician training, auditability of AI outputs, defined mechanisms for human oversight, and institutional policies for responding to AI-related errors will therefore be important components of safe implementation. WHO specifically emphasizes human responsibility and accountability, and recommends clearly defining the tasks that an AI system can perform and the conditions under which it can be appropriately used.

Regulatory requirements represent a further barrier to widespread clinical implementation. AI systems intended to support diagnosis, treatment, or other medical decisions may fall within medical-device or software-as-a-medical-device regulatory frameworks, depending on their intended use and jurisdiction. Regulatory assessment increasingly considers not only initial diagnostic performance but also the safety and effectiveness of the system across its lifecycle, including development, validation, deployment, monitoring, maintenance, and modification. For example, the U.S. Food and Drug Administration has issued recommendations addressing lifecycle management and marketing submissions for AI-enabled device software, and emphasizes risk management throughout the total product lifecycle. The FDA also identifies cybersecurity as an important consideration for medical devices containing AI/ML functions.

Regulatory considerations are particularly important for continuously updated or adaptive AI systems. A model that changes following software updates, new training data, or changes in its underlying algorithm may require reassessment to ensure that performance remains within the intended use and validated operating conditions. Consequently, future OMFS AI studies should provide sufficient information regarding the intended clinical use, training population, validation population, imaging environment, model version, update strategy, and monitoring procedures to permit meaningful assessment of safety and generalizability. International guidance increasingly emphasizes good machine learning practice, lifecycle management, external validation, transparency, cybersecurity, and ongoing performance monitoring rather than evaluation based solely on a single retrospective test dataset.

Taken together, these considerations indicate that successful implementation of AI in OMFS requires more than just high diagnostic accuracy. Clinical translation will depend on a combination of robust external validation, interpretable and clinically meaningful outputs, secure handling of patient data, seamless integration into PACS and existing clinical workflows, clearly defined human oversight and accountability, and compliance with applicable regulatory requirements. Future research should therefore evaluate AI systems not only in terms of accuracy and discrimination but also in prospective clinical environments, including workflow efficiency, clinician interaction, patient safety, usability, fairness, and real-world clinical outcomes.

### 4.2. Areas for Improvement in AI Applications in Oral and Maxillofacial Surgery

Despite significant advancements, several aspects of the use of AI in oral and maxillofacial surgery (OMFS) remain in need of improvement:

- Standardization and Clinical Workflow Integration: AI systems should be developed with interoperability with existing imaging infrastructure and PACS-based workflows in mind. Future studies should assess not only technical performance but also processing time, usability, clinician interaction, frequency of overrides, and the effect of AI implementation on diagnostic and surgical-planning workflows.
- Explainability and Human Oversight: Future AI systems should provide clinically interpretable outputs where feasible, and should clearly communicate uncertainty and limitations. Explainable-AI methods should themselves be validated, rather than assumed to provide reliable explanations. AI-generated recommendations should remain subject to appropriate clinician review and should complement, rather than replace, professional judgment.
- Prospective and Real-World Validation: Most currently available evidence is derived from retrospective, single-center datasets. Prospective multicenter studies and independent external validation are required to determine whether reported performance is maintained across different patient populations, imaging devices, acquisition protocols, disease prevalence, and clinical environments.
- Patient Privacy and Data Governance: Development and deployment should incorporate appropriate de-identification, secure data storage and transfer, access controls, cybersecurity measures, and compliance with applicable data-protection and consent requirements.
- Regulatory and Medico-Legal Frameworks: Clinical implementation requires clearly defined intended use, evidence of safety and effectiveness, lifecycle monitoring, documentation of model updates, and defined responsibilities among clinicians, healthcare institutions, developers, and manufacturers. Regulatory requirements should be considered from the early stages of AI development rather than only after technical validation.
- Surgical Planning Automation: Although AI can assist with anatomical landmark detection and procedural time prediction, further research is required before automated systems can reliably generate or recommend surgical plans. Such applications should undergo rigorous clinical validation and retain meaningful human supervision.
- Real-Time Intraoperative AI Guidance: Integration of AI with navigation systems, augmented reality, and other intraoperative technologies may eventually support surgical precision. However, these applications require prospective evaluation of accuracy, latency, usability, safety, and effects on clinical outcomes.
- Postoperative Monitoring and Outcome Prediction: AI tools capable of monitoring healing trajectories and predicting complications such as infection, nerve injury, or delayed bone healing warrant further investigation, particularly using prospectively collected multicenter datasets.
- AI for Rare Pathologies and Complex Patients: Current datasets are often dominated by common conditions and relatively standardized imaging examinations. Future models should be evaluated in patients with craniofacial anomalies, syndromic conditions, unusual pathology, previous surgery, and other clinically complex presentations.
- Ethical and Educational Integration: As AI becomes increasingly incorporated into OMFS, clinicians and trainees should receive education regarding appropriate interpretation of AI outputs, limitations of model performance, automation bias, data privacy, cybersecurity, and the professional responsibilities associated with AI-assisted decision-making.

Despite the considerable potential of AI in oral and maxillofacial surgery, implementation may be limited by differences in institutional resources, including access to compatible imaging equipment, computational infrastructure, software, and technical support. In addition, variability in CBCT acquisition parameters, reconstruction protocols, image quality, and scanner characteristics may affect model performance and generalizability across institutions. Therefore, future research should consider these practical and technical challenges when evaluating the transferability and clinical implementation of AI systems.

## 5. Conclusions and Recommendations

Artificial intelligence is increasingly being integrated into oral and maxillofacial surgery, with promising applications in anatomical landmark detection, diagnosis of complex conditions such as MRONJ, surgical planning, and prediction of surgical outcomes. Several studies have reported high performance of AI, but these should be interpreted cautiously, as most of these studies were retrospective, often single-center, and based on predefined imaging protocols with limited external validation. Reported performance may therefore not translate directly to routine clinical practice where patient populations, imaging protocols, and clinical workflows are more heterogeneous. Comparisons with human observers should consequently be considered study-specific and should not be interpreted as evidence that AI can replace expert clinical judgment.

### Recommendations

1. Future research should focus on large-scale, multicenter studies with diverse patient populations to validate AI performance across clinical settings and demographics.
2. AI tools should evolve beyond diagnostics into fully integrated decision-support systems that assist in surgical planning, identify intraoperative risks, and predict postoperative outcomes.
3. Efforts must be made to improve the interpretability of AI models to foster clinician trust and support regulatory approval.
4. Dental and surgical training programs should begin integrating AI practical applications into their curricula to prepare clinicians for AI-augmented practice.
5. Clear guidelines should be established to regulate the use of AI in dentistry, including standards for data privacy, accountability, and quality assurance.
6. Future models should be trained and tested on rare diseases, craniofacial anomalies, and syndromic cases to extend AI’s utility beyond routine care.

By following these recommendations, the dental and surgical communities can use the full potential of AI to enhance diagnostic accuracy, improve treatment outcomes, and elevate the standard of care in oral and maxillofacial surgery.

## Data Availability

All data generated or analyzed during this study are included in this published article and Supplementary Information files.

## Supplementary Materials

The following supporting information can be downloaded at: https://www.mdpi.com/article/10.3390/dj14100668/s1, Table S1. AI in the topics of OMFS—distribution of articles. Table S2. Analysis of reviewed articles and their contribution. Table S3. A traffic-light (risk of bias) table.
