# Supplementary material for: Artificial Intelligence Applications in Oral and Maxillofacial Surgery—A Narrative Review
Source: Dent J (Basel). 2026 Oct 13;14(10):668. doi: 10.3390/dj14100668 (PMC13703306; doi:10.3390/dj14100668)
Supplement: Supplementary file 1 [file dentistry-14-00668-s001.zip › dentistry-4499750-supplementary.pdf]

Table S1. Ai in the topics of OMFS- distribution of articles.

Subject	Number of articles
Anatomical structures identification	2
Dental implants	3
Fracture in the jaw and facial bones	2
Lesions +Cancer	9
Impacted Teeth	6
Mronj	2
Ortho-surgery	2
Osteoartritis+TMJ	3
Others including soft tissue	8
**Sum**	**37**

Table S2. Analysis of reviewed articles and their contribution.

Study	Algorithm Used	Study Factor	Modality	Number of Input Data	Performance	Comparison	Outcome
Choi et al (2022) (12)	Deep convolutional neural network model	relationship between the mandibular third molar and the inferior alveolar nerve	panoramic	571	Accuracy of 72.32% for contact Accuracy of 76% for buccolingual	Oral and Maxillofacial Surgery (OMFS) Specialists	The accuracy was higher than the OMFS
Evardsen et al 2022 (13)	EfficientDet DO model, Faster R-CNN, RetinaNet, CenterNet,	Automatic detection of the mental foramen for estimating mandibular cortical width	panoramic	5197	Accuracy of 79%	4 different models Faster R-CNN, RetinaNet, CenterNet, EfficientDet-D0	EfficientDet-D0 clearly performed better in terms of average precision.
Bayradkar et al 2021(14)	the deep convolutional neural network (Diagnocat inc	To evaluate the success of the artificial intelligence (AI) system in implant planning	three-dimensional cone-beam computed tomography(CBCT)	428-493 images	Accuracy of 72.2% for canals, 66.4% for sinuses/fossae.	Maxillofacial radiologists and AI	The results of the study were not satisfying for clinical use
Oh at al 2023(15)	ResNet-18,34,50 , DenseNet-121,201, MobileNet-V2 , and MobileNet-V3	prediction of osseointegration for dental implant	plain radiography	Group 1 (338 patients, 591 dental implants) Group 2 (242 patients, 615 dental implants	Accuracy of 80%	4 AI tools	osseointegration of dental implants can be predicted to some extent through deep learning using plain radiography

Study	Algorithm Used	Study Factor	Modality	Number of Input Data	Performance	Comparison	Outcome
Kim et al 2021(16)	DCNN	detection and classification of DI (dental implant) facture	Panoramic and perapical X rays	21,398 DIs from two dental hospitals, with 251 intact and 194 fractured DI images.	Accuracy of 80%	VGGNet-19, GoogLeNet Inception-v3, and an automated DCNN.	DCNNs, particularly automated architectures with periapical images, are effective in detecting and classifying fractured DIs
Shanavazi et al(2023)17	Faster Region-Based Convolutional Neural Network (Faster R-CNN).	Detection of mandibular fractures on	Panoramic X rays	190 panoramic images sourced from four different institutions.	accuracy of 91.67%	Comparing AI to general dentists	deep learning framework could serve as a valuable tool in diagnosing mandibular trauma
Son DM et al (2021)18	YOLO v4 (You Only Look Once)	Detection of mandibular fractures	panoramic radiographs	5621 patients	Accuracy of 0.935 the highest sensitivity was achieved for condyle fractures (55 out of 61	Comparing two approaches: one based on the shape of fractures (linear and shear) and another on anatomical regions (e.g., symphysis, body, angle)	The method could assist radiologists by providing a reliable tool for the diagnosis of mandibular fractures
Vinayahalingam S et al (2022)(19)	Faster R-CNN	Detection of mandibular fractures	Panoramic x rays	5621 patients	Accuracy of 0.947 – 0.977	Fractures versus non fractures	AI may help to reduce misdiagnosis of mandibular fractures

Study	Algorithm Used	Study Factor	Modality	Number of Input Data	Performance	Comparison	Outcome
Chai et al 2022 (20)	convolutional neural network	Preoperative diagnosis of ameloblastoma versus keratocyst	CBCT	350 X rays	Accuracy of 87.2%, to Ai and 66% for senior OMFS 58% for junior OMFS	Comparing Ai to OMFS	The AI was able to differentiate these two lesions with better diagnostic accuracy than clinical surgeons
Ariji E. et al 2019 (21)	deep neural network Detect Net	detect and classify radiolucent lesions in mandible.	panoramic radiographs	210 images	Sensitivity of 0.88	Two testing data sets	High sensitivity of detection and classification occurred for dentigerous cysts.
Lee et al et al 2020(22)	deep convolutional neural network	evaluate the detection and diagnosis of three types of odontogenic cystic lesions (	dental panoramic radiography and cone beam computed tomographic	2,126 images, 1,140 (53.6%) panoramic and 986 (46.4%) CBCT images.	CBCT sensitivity = 96.1%, specificity = 77.1%), greater than panoramic images (AUC = 0.847, sensitivity = 88.2%, specificity = 77.0%	Cbct versus panoramic x rays	deep convolutional neural network architecture trained with CBCT images achieved higher diagnostic performance than with panoramic images
Poedjiastoeti W, et al 2018 (23)	deep convolutional neural network	detection of ameloblastomas and KCOTs.	Panoramic images	500 X rays	sensitivity, specificity, accuracy of above 80% for CNN and for OMFS	CNN versus OMFS	Similar results but shorter time for diagnosis by AI

Study	Algorithm Used	Study Factor	Modality	Number of Input Data	Performance	Comparison	Outcome
Romeo V, et al 2020(24)	radiomic machine learning	to prediction of tumor grade) and nodal status in patients with oropharyngeal and oral cavity squamous-cell carcinoma (SCC).	Contrast-enhanced CT images	40 patients	Accuracy of above 90%	different machine learned algorithms	A radiomic ML approach applied to primary tumor lesions is able to predict tumor grade and nodal metastasis
Ha EG et al 2023(25)	EfficientDet model	Classification of retention pseudocysts in the maxillary sinuses	Panoramic X ray	213 patients 426 sinuses	Accuracy of 81% for pseudocysts, 90% for cysts and 98% for healthy sinuses	Cysts pseudocysts and healthy sinuses	The model proposed excellent diagnostic performance
Murata R et al 2020(26)	deep learning system	Diagnosis of maxillary sinus lesions	Panoramic X ray	501 patients	Accuracies for diagnosis maxillary sinusitis were 90-91%, for cysts of the maxillary sinus regions, these values were 97-100%,	Healthy sinuses, inflamed sinuses, cysts sinuses	Deep learning could detect the maxillary sinuses and identify maxillary sinusitis and cysts of the maxillary sinus regions
Ohashi (2016)(27)	CAD system	Diagnosis of sinusitis	Panoramic X rays	300 cases	CAD system provided sensitivity of 77.6%, specificity of 69.4% and accuracy of 73.5%.	Inexperience dentists experts of oral and maxillofacial radiology cuputer aidedc	Cad support helped inexperience dentists

Study	Algorithm Used	Study Factor	Modality	Number of Input Data	Performance	Comparison	Outcome
Kowana R et al (2020)(28)	Deep learning	Identify maxillary sinus lesions	panoramic radiography	1174 cases			
Vollmer a et al (29)	VGG16, InceptionV3, MobileNetV2, EfficientNet, and ResNet50	to predict OAC versus non-OAC	panoramic radiography	300 cases	Ai presented low sensitivity for prediction false negative of 57-95%	Ai versus experts	It is not possible to predict OAC from preoperative panoramic X rays
Orhan K et al 2021(30)	Ai for evaluation for third molars	Evaluation of 3rd molars :Impaction detection, Impacted tooth numbers, root/canal numbers of teeth, relationship with adjacent anatomical structures	CBCT	65 patients 130 3rd molars	86% detection by AI, 78% for number of roots setermination, 68% or number of canals detection	Ai and human observer	Ai showed high accuracy values in the detection of impacted third molar teeth and their relationship to anatomical structures.
Dai X et al 2023(31)	deep mesiodens localization network (DMLnet)	Mesiodens detection and localization	Panoramic X ray	Not mentioned	Describing the way of one shot for analysis	Ai	The model showed a potential for the automated detection of mesiodens, providing valuable assistance to dentists in accurate diagnosis.
Ha eg et al (2021)(32)	DCNN	Mesiodens detection	Panoramic X ray	612 patients	High accuracy of detection for al dentition types (around 90%)	3 dentition groups	Could serve use to detect mesiodens on panoramic radiographs of all dentition types

Study	Algorithm used	Study Factor	modality	Number of Input Data	performance	comparison	outcome
Kim et al (2022)(33)	DeeplabV3plus Inception-resnet-v2	Mesiodens detection	Panoramic X ray	988 patients	High accuracy of detection of mesiodens	Panoramic x rays with mesiodens and without mesiodens	a fully automated process for diagnosing mesiodens using only panoramic radiographs. panoramic radiographs
Kowada et al(2020)(34)	AlexNet, VGG-16, and DetectNet	classifying maxillary impacted supernumerary teeth.	Panoramic X ray	550 panoramic x rays	DetectNet produced the highest values of diagnostic efficacy	3 deep learning systems	DetectNet would be suitable for automatic detection of supernumerary teeth
Ahn et al (2021)(35)	SqueezeNet, ResNet-18, ResNet-101, and Inception-ResNet-V2	classify mesiodens in primary or mixed dentition	Panoramic X ray	550 panoramic x rays	ResNet-101 and Inception-ResNet-V2 were higher than 90%	3 deep learning systems	Deep-learning technologies may help unexperienced clinicians in more accurate and faster diagnosis.
Kwack et al (2023) (36)		Algorithm to predict MRONJ in patients with oeteoporosis undergoing dental implants and extraction	Clinical symptoms	340 patients	Accuracy of 0.8283	6 different algorithms	The models can help predict MRONJ occurrence in patients with osteoporosis
Coban E et al (2024)(37)			questionaire		use of the artificial intelligence platform may assist in patients gaining a fundamental understanding of MRONJ		The artificial intelligence model has generated responses of moderate quality to questions about MRONJ.

study	Algorithm used	Study factor	modality	Number of Input Data	performance	comparison	outcome
Whilkat et al (2021)(38)	(BrainLAB AG	Analyze the size of MRONJ and facilitate diagnosis	3D imaging via spiral or cone beam computed tomography w	164 patients		Different parameters to size of MRONJ defect	3D image is recommended for MRONJ diagnosis the exact defect volume serves as a decision-making aid with regard to the choice of therapy,
Shoukri et al 2024(39)	Neural network	Shape analysis of condylar morphology biomarkers	CBCt and blood and correlations to saliva samples	70 patients	significant correlations among variations in protein expression levels, clinical symptoms, and condylar surface morphology	Clinicians and AI	3-dimensional variability in condylar morphology can be phenotyped by the neural network
Choi et al 2021(40)	Res net	Osteoarthritis grades	Panoramic x rays	1189 patients	Ai sensitivity was quivalent to that of an expert	Ai and maxillofacial experts	AI can play an important role in primary diagnosis of TMJOA from OPGs in most general practice clinics where OMFR experts or CT are not available.

study	Algorithm used	Study factor	modality	Number of input data	performance	comparison	outcome
Jung et al(2023)(41)	Resnet152 and EfficientNet-B7 as transfer learning models.	Normal versus osteoarthritis in panoramic X rays	Panoramic X rays	858	Ai exhibited accuracy of 0.87 0.88 in classification of osteoarthritis	Dentistst TMD specialists and AI	Ai can be effectively applied by dentists as a screening diagnostic tool for TMJ-OA.
Song et al (2022)(42)	R-cnn model	Soft tissue calcifications	Panoramic x rays	352 images	AI reduced the total reading time of the specialists- and improved the accuracy reading of GP the detection of sialolith. Diagnosis of Calcifications in the carotid is higher than that in lymph node.	General dentists, maxillofacial radiologists and AI	AI helped to improve the diagnostic accuracy of the GD group,
Katz et al (2022)(43)	Faster R-CNN	Detection of atherosclerotic plaques	Panoramic X rays	66 images	75% sensitivity 83% acuracy	Ai only with change of the brightness and rotations	Novelty of AI for detection of plaques in the carotid
Lee A et al 2021(44)	Deep learning model for imaging	Diagnosis of stafne defect in mandible	Panoramic X ray	176 Stafne's bone cavities and 282 odontogenic cysts and tumors of the mandible	99.25% accuracy, 98.08% sensitivity, and 100% specificity	Stafne bone cavities and odontogenic cysts and tumors	The model showed high performance

study	Algorithm used	Study factor	modality	Number of input data	performance	comparison	outcome
Orhan et al (2022)(45)	CNN	Detection of pharyngeal airway on CBCT	CBCT	100 cases	No statistical difference between manual measurements and diagnocat measurements	Manual measurements and AI	AI algorithm is not precise as manual measurements due to limitation of x rays
ter Horst R et al (2021)(46)	Deep learning algorithm	Predict soft tissue profile after mandibular advancement	Cbct and 3D photographs	133 patients	a significant correlation between the defect volume and the MRONJ stage, the number of performed operations, and the therapy invasiveness.	Ai algorithm with tensor flow	Soft tissue prediction is a relevant option by DL algorithm
Tao T et al (2023)(47)	3 D U Net SE	Ai detection for available sites for palatal minimplants determination	CBCT	70 patients	Ai had high performance for thickness measuring and segmentation	Orthodontics and AI	Ai could be helpful for determination of available sites for mini implants
Kwon et al (2022)(49)	CNN+MLP	Assessing the required time for extraction of lower 3rd molar	Panoramic X rays	724	The combined model was good with high level of significance	Ai predicted time by actual exact time	The model predicted the exact time accurately
**study**	**Algorithm used**	**Study factor**	**modality**	**Number of input data**	**performance**	**comparison**	**outcome**

Jain A, Jadhav P (2026) 50	Five algorithms were compared, with Random Forest and XGBoost	Prediction of postoperative complications following 3rd molar extraction- retrospective	Panoramic X ray	472	Random Forest and XGBoost demonstrating superior predictive performance (AUC 0.91 and 0.89, respectively) compared with logistic regression (AUC 0.74).	Ai prediction and actual complications	artificial intelligence to provide clinically interpretable and individualized risk stratification
Trachoo V et al (2024)51	ResNet101V2 handled , RetinaNet Vision Transformer	Predicting the Difficulty Level of Removing the Impacted Mandibular Third Molar	Panoramic X ray	784 patients	ResNet101V2 model achieved a classification accuracy of 0.8671. RetinaNet model - exceptional detection performance, with a mean average precision of 0.9928. Vision Transformer model - an average accuracy of 0.7899 in predicting removal difficulty levels.		

Table S3. A traffic-light (risk of bias) table.

Study	Selection bias	Ground Truth	Validation	Overfitting risk	Overall
Choi et al.	● yellow	● green	● yellow	● yellow	● yellow
Edvardsen et al.	● dark green	● yellow	● light green	● light green	● light green
Bayrakdar et al.	● yellow	● dark green	● yellow	● yellow	● yellow
Oh et al.	● red	● yellow	● yellow	● red	● red
Lee et al.	● yellow	● light green	● yellow	● yellow	● yellow
Shahnavazi et al	● yellow	● light green	● red	● red	● red
Son et al.	● yellow	● light green	● yellow	● yellow	● yellow
Vinayahalingam et al	● light green	● light green	● dark green	● yellow	● dark green
Chai et al.	● yellow	● light green	● yellow	● yellow	● yellow
Ariji et al.	● yellow	● yellow	● red	● red	● red
Lee et al.	● yellow	● light green	● yellow	● yellow	● yellow
Poedjiastoeti et al.	● red	● yellow	● red	● red	● red
Romeo et al.	● yellow	● light green	● yellow	● red	● red
Ha et al.	● yellow	● yellow	● yellow	● yellow	● yellow
Murata et al.	● yellow	● yellow	● red	● red	● red

Study	Selection bias	Ground Truth	Validation	Overfitting risk	Overall
Ohashi et al.	🔴	🟡	🔴	🔴	🔴
Kuwana et al.	🟡	🟡	🟡	🟡	🟡
Vollmer et al.	🟡	🟢	🟡	🔴	🔴
Orhan et al.	🟡	🟢	🟡	🟡	🟡
Dai et al.	🟡	🟢	🟡	🟡	🟡
Ha et al.	🟡	🟢	🟡	🟡	🟡
Kim et al.	🟡	🟢	🟡	🟡	🟡
Kuwada et al	🟡	🟢	🟡	🟡	🟡
Ahn et al.	🟡	🟢	🟡	🟡	🟡
Keack & Park	🔴	🟡	🟡	🔴	🔴
Coban & Altay					
Wilkat et al	🟡	🟡	🟡	🟡	🟡
Shoukri et al	🟡	🟢	🟡	🟡	🟡
Choi et al	🟡	🟡	🟡	🟡	🟡
Jung	🟡	🟡	🟡	🟡	🟡
Song	🟡	🟢	🟡	🟡	🟡
Katz	🟡	🟡	🔴	🔴	🔴

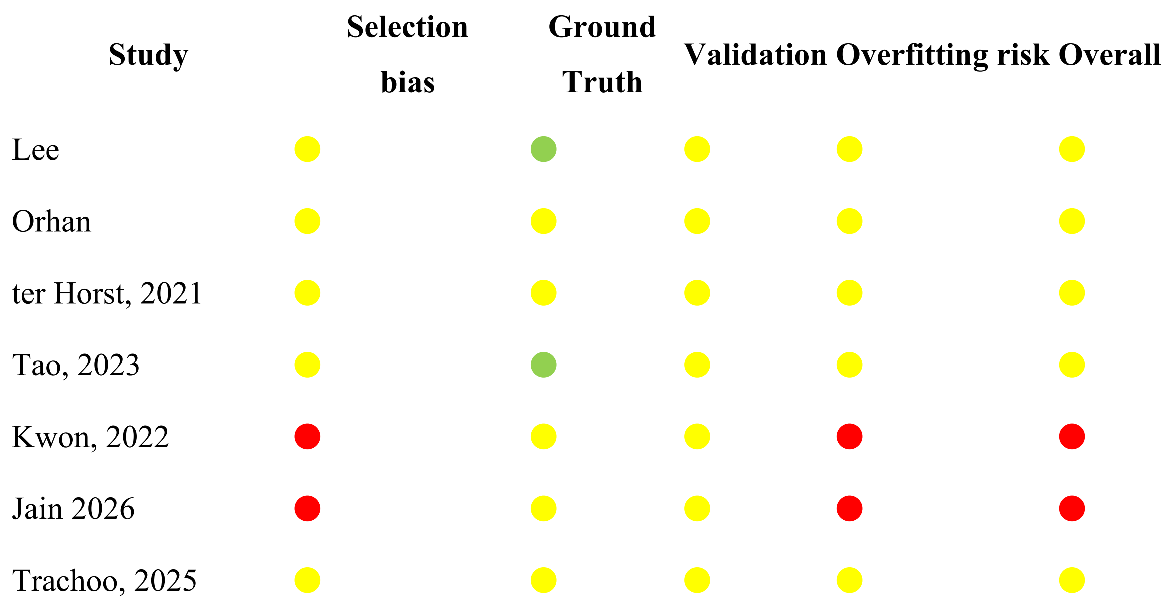
Study
Selection bias
Ground Truth
Validation
Overfitting risk
Overall
Lee
Orhan
ter Horst, 2021
Tao, 2023
Kwon, 2022
Jain 2026
Trachoo, 2025
